# The Role of SATB1 in Tumour Progression and Metastasis

**DOI:** 10.3390/ijms20174156

**Published:** 2019-08-25

**Authors:** Natalia Glatzel-Plucińska, Aleksandra Piotrowska, Piotr Dzięgiel, Marzenna Podhorska-Okołów

**Affiliations:** 1 Division of Histology and Embryology, Department of Human Morphology and Embryology, Wroclaw Medical University, 50-368 Wroclaw, Poland; 2 Department of Physiotherapy, Wroclaw University School of Physical Education, 51-612 Wroclaw, Poland; 3 Division of Ultrastructure Research, Wroclaw Medical University, 50-368 Wroclaw, Poland

**Keywords:** SATB1, Special AT-Rich Binding Protein 1, EMT, epidermal-mesenchymal transition, metastasis, cancer

## Abstract

Carcinogenesis is a long-drawn, multistep process, in which metastatic spread is an unequivocal hallmark of a poor prognosis. The progression and dissemination of epithelial cancers is commonly thought to rely on the epidermal-mesenchymal transition (EMT) process. During EMT, epithelial cells lose their junctions and apical-basal polarity, and they acquire a mesenchymal phenotype with its migratory and invasive capabilities. One of the proteins involved in cancer progression and EMT may be SATB1 (Special AT-Rich Binding Protein 1)—a chromatin organiser and a global transcriptional regulator. SATB1 organizes chromatin into spatial loops, providing a “docking site” necessary for the binding of further transcription factors and chromatin modifying enzymes. SATB1 has the ability to regulate whole sets of genes, even those located on distant chromosomes. SATB1 was found to be overexpressed in numerous malignancies, including lymphomas, breast, colorectal, prostate, liver, bladder and ovarian cancers. In the solid tumours, an elevated SATB1 level was observed to be associated with an aggressive phenotype, presence of lymph node, distant metastases, and a poor prognosis. In this review, we briefly describe the prognostic significance of SATB1 expression in most common human cancers, and analyse its impact on EMT and metastasis.

## 1. Introduction

Cancer is the second leading cause of death worldwide, accounting for more than 9 million deaths annually [1,2]. Although cancer-related mortality has slowly been in decline for over 25 years, worldwide about 1 in 6 deaths is still due to malignant tumours [3]. Neoplasms are often considered as a developed-world problem, but 57% of all cases (excluding non-melanoma skin cancer) occur in less-developed countries [4]. This highlights cancer as a global health concern, independent of nationality and income levels. 

It is well known that most cancer-related deaths are caused not by a primary tumour itself, but by its metastases to distant organs, especially to the brain, liver, lungs and bones [5,6,7]. Carcinogenesis is a long-drawn, multistep process, in which metastatic spread is an unequivocal hallmark of a poor prognosis. The exact mechanism of cancer cell dissemination seems to be very complex and has not been fully understood yet. The two most substantial theories regarding metastasis were proposed at the turn of the 19th and 20th century by Stephen Paget and James Ewing [8,9]. Paget, in his “seed and soil” hypothesis, assumed that tumour cells metastasize specifically only to the sites where the environment is favourable [5,8]. Ewing, in turn, stated that metastasis is determined purely by the vasculature anatomy and blood flow mechanics, and that cancer cells, after entering the bloodstream, will spread randomly to the first organ encountered [5,9]. At present, it is known that metastasis mechanisms are much more complicated and depend on many factors, including biochemical, anatomical and mechanical ones, thus making it impossible to explain this process with one simple, universal theory.

**Figure 1:**
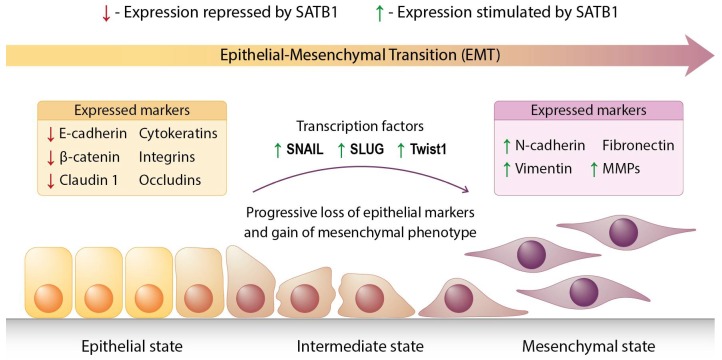
Diagram depicting the epithelial-mesenchymal transition process. Cobblestone-like epithelial cells (“Epithelial state”, on the left) gradually lose their junctions and apical-basal polarity, and become spindle-shaped and invasive (“Mesenchymal state”, on the right). The epithelial and mesenchymal markers commonly used to describe EMT progression are listed above the specific cells. The transition process is mediated by the three main transcription factors: SNAIL, SLUG, and Twist1. The impact of SATB1 on the expression of particular factors is indicated by the red (expression repressed) or green (expression stimulated) arrows.

The dissemination of epithelial cancers is commonly thought to rely on the epidermal-mesenchymal transition (EMT) process [10]. During EMT, epithelial cells lose their junctions and apical-basal polarity, and they acquire a mesenchymal phenotype with its migratory and invasive capabilities [11]; Figure 1. It is commonly assumed that in malignant cells the uncontrolled activation of EMT-related traits occurs due to a gradual loss of genetic stability and the accumulation of mutations during cancer progression [12]. Nevertheless, there are evidences that some cancer cells may acquire the ability to spread and metastasize even before the full malignant transformation, suggesting that EMT may be an early event during carcinogenesis [13]. Therefore, it is likely that cancer cells could acquire their metastatic potential right after the first oncogenic mutations.

For over 50 years carcinogenesis was thought to rely on the sequential accumulation of gene mutations. It was assumed that 6 or more genetic alterations were required to initiate the most common types of cancer [14,15]. However, recent studies have shown that in order to trigger the pathways leading to advanced cancers of lung and colon, only 2 or 3 mutations are sufficient [16]. After its initiation, cancer gradually evolves, gaining new mutations and aberrant gene expression patterns. The analysis of typical solid tumours revealed so many genetic alterations that it would be almost impossible to identify the most important “driver” ones. Moreover, epigenetic modifications, gene fusions and chromosomal translocations additionally complicate our understanding of the molecular basis of cancer. The process of cancer progression is very complex, but one should not forget that its starting point may just be a few genetic disorders. The important question is whether it is possible for mutations or changes in the expression of only a single gene to lead to the activation of other cancer-promoting mechanisms, including EMT. In 2008, Han et al. described a unique protein that is able to promote tumour progression and metastasis by globally changing the transcriptional profiles of hundreds of genes [17]. It was Special AT-Rich Binding Protein 1 (SATB1), a powerful transcription factor with wide regulatory abilities.

### SATB1 Protein

SATB1 is a chromatin organiser and a global transcriptional regulator described for the first time by Dickinson et al. in 1992 [18]. It binds to specific AT-rich motifs of double-stranded DNA, and it organizes chromatin into spatial loops [19,20]. These AT-rich sequences are called base-unpairing regions (BURs), and they may be found every 40,000 DNA base pairs [21]. SATB1 bound to BURs provides a “docking site”, necessary for the binding of further transcription factors and chromatin modifying enzymes [21]. It also maintains epigenomic modifications and proper nucleosome positioning [21]. Therefore, SATB1 has the ability to regulate whole sets of genes, even those located on distant chromosomes [19,20]. SATB1 interactions with transcription activators and repressors are determined by its post-transcriptional modifications like phosphorylation or acetylation [22,23]. It was shown that STAB1 can also be regulated by numerous microRNAs, including miR-191, miR-155, miR-448, miR-7, miR-302a-3p and miR-21-5p [24,25,26,27,28,29,30]. SATB1 regulates gene expression on a tissue-specific manner—it binds to distinct genomic regions and regulates different sets of genes depending on the cell type [31]. It has been demonstrated that SATB1-dependent gene sets in breast cancer cells and mouse primary keratinocytes have only minimal overlap [31]. In breast cancer cells, SATB1 stimulated the expression of genes promoting metastasis, cell proliferation, angiogenesis and cell adhesion, whereas in the primary keratinocytes, it mainly regulated genes responsible for cell differentiation and development, as well as those coding keratin-associated proteins [31]. It is estimated that SATB1 may influence the expression of more than 10% of human genes [22,23].

Physiologically, a high SATB1 level is observed in embryonic stem cells and numerous adult progenitor cells like ameloblasts and osteoblasts [19,32,33]. SATB1 takes part in embryonic development, differentiation and maturation of thymocytes and skin epithelial cells, as well as other processes that require rapid changes in the cell phenotype [19]. Its expression is essential for T-cells maturation—in SATB1-*null* mice almost all thymocytes were blocked at the CD4+/CD8+ double positive stage, and the mice died at the age of 3 weeks [32]. An appropriate SATB1 level was also shown to be necessary for a proper lung development during embryogenesis [34].

Besides the normal, physiological processes, SATB1 was found to be overexpressed in numerous malignancies, including lymphomas, breast, colorectal, prostate, liver, bladder and ovarian cancers, osteosarcoma and glioma [17,35,36,37,38,39,40,41]. In the solid tumours, its high level was observed to be associated with an aggressive phenotype and a poor patients’ prognosis [17,39,40,42,43,44,45]. Additionally, it has been shown that SATB1 may influence the EMT process and promote cancer metastasis [39,46,47,48]. Induction of the SATB1 expression was sufficient to transform cultured non-invasive cells into aggressive, tumorigenic ones [17]. Its depletion had a reverse effect: an SATB1 knockdown in highly aggressive cancer cells was demonstrated to be enough to restore their normal morphology and decrease their migration and invasion abilities [17,49,50]. These results could point to SATB1′s function as a specific trigger of a malignant phenotype, clearly contributing to carcinogenesis. In this review, we will consider the importance of SATB1′s expression in the progression of the five most common human neoplasms: cancers of the breast, lung, colorectum, prostate and stomach.

## 2. SATB1’s Role in Cancer Progression

### 2.1. Breast Cancer

The earliest and most comprehensive study concerning SATB1′s role in breast cancer progression was published in 2008 by Han et al. [17]. SATB1 and its mRNA were only detected in metastatic breast cancer cell lines, and their levels were correlated with the aggressiveness of the cells [17]. Moreover, the SATB1 protein was found to be overexpressed in breast cancer specimens as compared to adjacent non-malignant breast tissues, and the high level of its expression was associated with a poor degree of tumour differentiation [17]. These findings were further confirmed by Zhang and colleagues, who showed that SATB1 was abundantly expressed in breast cancer specimens, while its expression was almost undetectable in normal and being-changed tissues [51]. SATB1′s level increased gradually during the progression from non-malignant breast tissue, through cystic hyperplasia and precancerous lesions, to breast cancer at the end [51]. Moreover, SATB1′s overexpression was associated with positive HER-2 status, higher TNM stage, and the presence of lymph node metastasis [51]. An increased SATB1 protein level in breast cancer cases as compared to normal breast tissues, and its positive correlation with a higher histological grade and a positive HER-2 status were also further reported by Liu et al. [52]. Similarly, Wang and co-workers observed that SATB1′s expression positively correlated with the size and grade of the tumour, the presence of lymph node metastasis, the stage of the disease and the tumour ER status [53]. A positive relationship between the level of SATB1 and a poor degree of tumour differentiation was also demonstrated by Kobierzycki et al., but their results did not reach statistical significance [54].

A number of studies have found a significant association between SATB1′s expression and the metastatic potential and aggressiveness of breast cancer cells. In their pioneering work, Han et al. emphasized SATB1′s role as an important factor promoting mammary tumours’ growth and metastasis [17]. They demonstrated that siRNA-mediated SATB1 silencing in highly aggressive MDA-MB-231 breast cancer cells resulted in a significant reduction of their invasive capacity and prevented the formation of colonies [17]. Moreover, SATB1-depleted 

MDA-MB-231 cells formed far less metastatic nodules when injected in mice compared to the wild type ones [17]. The authors concluded that SATB1′s expression is necessary for the aggressive, highly metastatic phenotype of MDA-MB-231 cells. To support these findings, the researchers expressed SATB1 ectopically in the non-tumorigenic SKBR3 breast cancer cell line. The modified SKBR3 cells, after being injected in mice mammary glands, developed large, undifferentiated, highly vascularized tumours [17]. Further gene expression analysis revealed that SATB1-depleted MDA-MB-231 cells presented changes in the expression level of about 1000 genes mainly associated with cell adhesion, phosphatidylinositol signalling, cell cycle regulation and lung and bone metastasis [17]. Among the 231 Rosetta poor prognosis-associated genes [55], the expression of 63 of them was altered by SATB1 depletion [17]. Except for the upregulation of metastasis-promoting factors like Metastasin, VEGF B, metalloproteases and the Transforming Growth Factor β, SATB1 was also shown to impact the expression of EMT-related proteins [17]. SATB1 seemed to promote the mesenchymal phenotype of cancer cells by upregulating Vimentin and N-cadherin, as well as downregulating the key epidermal markers Claudin-1, β-catenin and E-cadherin [17]; [Table 1]. SATB1 depletion in MDA-MB-231 cells was found to reverse the EMT process and significantly change the phenotype of the cells, restoring their polarization and acinar-like morphology [17].

Despite using the same cell lines and methods, the results of Han et al. were not confirmed by Iorns et al. [56]. In the latter’s experiments, SATB1 silencing did not affect the aggressive phenotype of MDA-MB-231 cells [56]. Moreover, modifying SATB1′s expression did not alter the anchorage-independent cell proliferation and migration ability in cultured cells, and it did not affect tumour formation and metastasis in xenograft mouse models [56]. To date, the study conducted by Iorns et al. is the only one refuting that SATB1′s expression could promote the progression of breast cancer [56,57]. These findings were disputed by members of Han’s research team, who in a response paper carefully evaluated the methodology and cell lines used by Iorns et al., revealing numerous weaknesses like the heterogeneity of the used cell lines or the lack of specificity of the RNA probes used in the study [58].

Despite this, further studies have supported the results of Han et al. and their theory that SATB1′s expression is closely associated with the aggressive phenotype of breast cancer cells and may contribute to EMT. In 2010, Li et al. revealed that SATB1 plays an important role in the induction of chemotherapy-related EMT in breast cancer cells [26]. Another evidence of the interrelation between SATB1′s expression and EMT was described by Sun et al., who investigated the mechanism underlying the regulation of breast cancer stem cells’ (BCSC) population within tumours [59]. They found out that SATB1′s expression not only increased the number of BCSCs, but also stimulated the expression of Snail and Twist1—the most crucial EMT-associated transcription factors [59]; Table 1. Finally, Ma et al. revealed that the anti-metastatic activity of flavonoid baicalein in breast cancer cells may be caused by the inhibition of EMT via downregulation of SATB1 and the Wnt/β-catenin pathway [60]. These results are in line with the previous findings by Gao et al., who reported that baicalein inhibited the proliferation, migration and invasiveness of MDA-MB-231 cells by downregulating SATB1′s expression [61].

Many attempts have been made in order to define the prognostic value of SATB1′s expression in mammary tumours. Han and co-workers observed that a high SATB1 level was strongly associated with a shorter overall survival (OS) time of breast cancer patients [17]. However, these results were not confirmed by Ions et al., who showed the lack of association between *SATB1* mRNA expression and a decreased OS of primary breast cancer patients [56]. Further studies also demonstrated ambiguous results. Patani et al. observed that a higher *SATB1*′s expression was associated with a shorter OS, but the results lacked statistical significance [62]. Hanker and colleagues analysed *SATB1*′s expression using Affymetrix microarrays in a cohort of more than 2000 breast cancer samples [63]. They observed no relationship between *SATB1*′s expression and patients’ OS in ER-negative breast cancers, while in ER-positive tumours, a high *SATB1*′s transcript level was found to be a predictor of better prognosis [63]. Contrastingly, Liu et al. showed that SATB1 was an independent negative prognostic factor in breast cancer patients [52]. In their study, the presence of SATB1 protein predisposed to a worse prognosis, and the risk exposure was approximately 2.5 times higher than in the SATB1 negative group [52]. The prognostic significance of SATB1 was also confirmed in a study by Laurinavicius et al., who examined immunophenotypes of hormone receptor positive invasive ductal breast cancer specimens [64]. They observed that a high Ki67/SATB1 ratio was an independent predictor of worse OS [64]. Although SATB1 did not reach statistical significance as a single prognostic factor, there was a visible trend of association between a higher SATB1′s expression and poor OS [64]. However, these results were not confirmed by Kobierzycki et al. [65].

In 2016, Pan and colleagues performed a meta-analysis to summarize the clinical and prognostic relevance of SATB1′s expression in mammary tumours [57]. They analysed 10 studies involving a total of 5185 patients, and the showed that SATB1′s expression positively correlated with breast cancer progression [57]. High SATB1 levels were related to the presence of lymph node metastasis and a higher TNM stage, two factors that are associated with a reduced life expectancy in breast cancer patients [57]. Therefore, SATB1 might be a novel predictive factor in breast tumours [57].

### 2.2. Lung Cancer

The impact of SATB1′s expression on lung cancer progression seems to be strictly dependent on the exact histological type of the tumour, which is why the distinction between different subtypes is necessary. Lung cancers are typically divided into two main groups based on their histological structure and molecular basis: small-cell lung carcinomas (SCLCs; 20% of cases) and non-small cell lung carcinomas (NSCLCs; 80% of cases) [86]. Among NSCLCs, further histological subtypes can be distinguished, the most common ones being the adenocarcinoma (AC) and the squamous cell carcinoma (SCC) [86].

**Figure 2:**
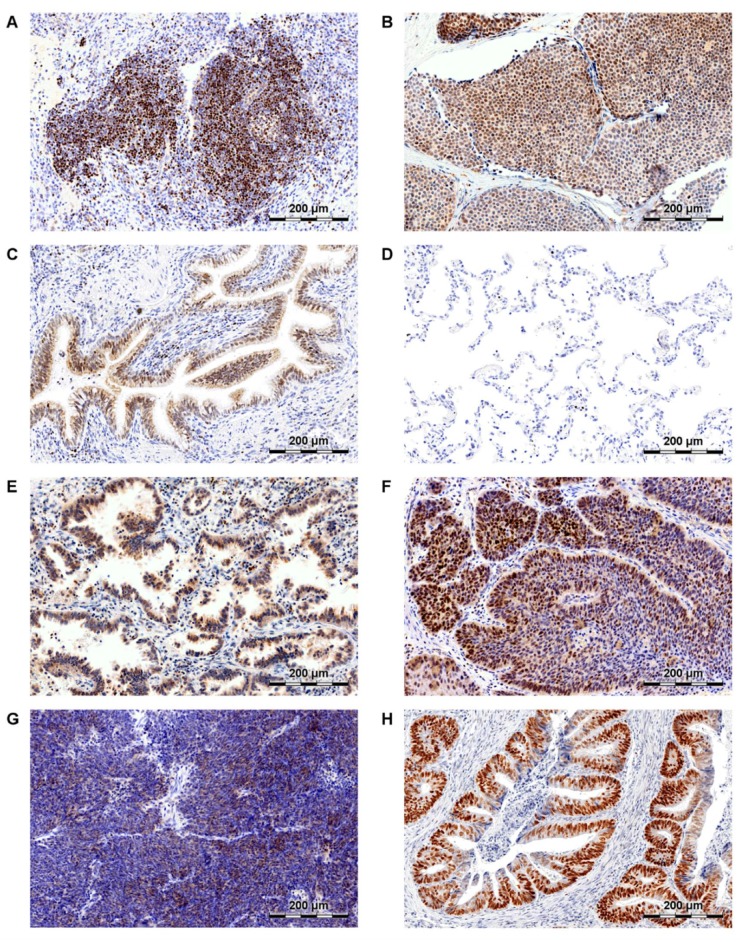
SATB1′s expression in human lymphocytes (**A**), breast cancer (**B**), normal airway epithelium (**C**), normal lung alveoli (**D**), lung adenocarcinoma (AC; **E**), lung squamous cell carcinoma (SCC; **F**), small cell lung carcinoma (SCLC; **G**) and colorectal cancer (CRC; **H**).

The role of SATB1 in lung cancer has been researched in only a few studies concerning mainly NSCLCs. In 2009, Zhou et al. revealed that *SATB1* mRNA was overexpressed in NSCLC samples when compared to non-malignant lung tissues [87]. However, gene expression data from three microarray studies did not confirm these findings. On the contrary, *SATB1* mRNA expression was found to be significantly down-regulated in cancer cells in comparison to normal lung samples [88,89,90]. These discrepancies could have been caused by the varied SATB1 expression patterns in the non-malignant lung tissues. As we have shown in our recent study [91], SATB1 level was relatively high in the normal bronchial epithelium, whereas its expression was almost undetectable in the lung alveoli (Figure 2C,D, respectively). Zhou and colleagues did not specify what kind of tissue was used as a “normal lung” control in their experiments.

To date, the most extensive study analysing SATB1′s impact on lung cancer progression was published in 2012 by Selinger et al. [92]. In this study, SATB1′s expression was found to be significantly decreased in NSCLC samples as compared to normal bronchial tissues [92]. Moreover, a high SATB1 level was associated with the SCC histological type, poor tumour differentiation and an early stage of the disease [92]. These results were further confirmed by several groups who analysed SATB1′s expression in AC and SCC subtypes separately. Huang et al. noticed an increased SATB1 level in AC specimens as compared to adjacent non-malignant lung tissues [49]. However, the authors did not determine what kind of tissue served as a control, the pulmonary alveoli themselves or in association with the bronchioles. SATB1′s expression was visibly related to a poor degree of tumour differentiation and an advanced TNM stage [49]. In our recent study, we observed diverse SATB1 expression patterns depending on the histological type of NSCLC tumour [91]. SATB1 level was found to be significantly higher in SCCs in comparison to AC specimens, confirming previous findings made by Selinger et al. [91,92]. A similar relationship was also observed in SCC and AC cell lines [91]. Moreover, in AC clinical samples SATB1′s expression was associated with a poor degree of tumour differentiation, whereas in the SCC subtype the level of SATB1 was increased in well differentiated tumours [91]. Additionally, a positive correlation between the expression of SATB1 and the proliferative marker Ki67 was noticed in SCCs [91].

These initial results suggested that in AC an elevated SATB1 level may be associated with a more aggressive, malignant tumour phenotype. In order to be able to examine this relationship more thoroughly, Huang et al. silenced SATB1′s expression in a highly aggressive AC cell line—A549 [49]. SATB1′s knockdown significantly reduced cell proliferation, migration and invasion rates, and increased apoptosis [49]. Unfortunately, there are no reports available about the effect of SATB1′s depletion on SCC type cells.

There are only two studies that analyse the prognostic significance of SATB1′s expression in NSCLC. Selinger et al. revealed that a loss of SATB1′s expression was a negative prognostic factor for SCC (but not for AC) patients [92]. Additionally, a decreased SATB1 level was found to be associated with a shorter overall survival of NSCLC patients who had ever smoked [92]. Similarly, our research team observed [91] that an elevated SATB1′s expression was a positive prognostic factor for NSCLC patients (AC and SCC subtypes analysed together). However, we did not confirmed the prognostic value of SATB1′s expression in the SCC subtype (our results were on the verge of statistical significance) [91].

In SCLC, the impact of SATB1′s expression on the progression of the disease seems to be as ambiguous as in NSCLC. SCLC is an extremely aggressive kind of lung cancer, characterized by a rapid growth and an early spread to distant sites [93]. Despite the sensitivity to chemo- and radiotherapy, SCLC is a complex therapeutic problem due to its high recurrence rate [86]. The earliest study in which SCLC clinical samples were included was conducted by Selinger et al. [92]. While in NSCLC samples SATB1′s expression was observed to be lost as compared to normal bronchial epithelium, in SCLC cases it was relatively high [92]. There was actually no difference in SATB1′s expression between SCLC and non-malignant bronchial epithelium [92]. Due to the small number of samples (14 SCLC cases), no significant associations between the SATB1 level and the patients’ clinicopathological data were observed [92]. However, a high SATB1 level was found to be a predictor of better prognosis [92]. These results undoubtedly need validation with a larger experimental cohort, especially since the SATB1-negative group comprised only 2 patients. Furthermore, in 2013, Huang et al. observed SATB1′s overexpression in 29 SCLC samples as compared to paired adjacent normal tissues [93]. Unfortunately, the authors did not specify the exact kind of tissue used as a control, so it remains unclear whether it was pulmonary alveoli or bronchial epithelium.

Besides the experiments on SCLC clinical samples, Huang and colleagues also studied SATB1′s role in vitro by using a SATB1 loss-of-function model. They performed siRNA-mediated SATB1 silencing in the NCI-H446 SCLC cell line [93]. SATB1′s knockdown was observed to inhibit cells’ proliferation and invasion and to induce apoptosis [93]. SATB1-depleted cells displayed also morphological changes—they were smaller, rounder and less confluent than the control ones [93]. The authors concluded that SATB1 may be a promising target for novel SCLC therapies. However, their results and those obtained by Selinger et al. [92] seemed to be contradictory and clearly indicated the need of more comprehensive research on an extended group of SCLC patients.

### 2.3. Colorectal Cancer

The first study on the role of SATB1 in colorectal cancer (CRC) was published in 2011 by Meng et al. [94]. Their results indicated that SATB1 may play an important role in human rectal cancer progression [94]. The analysis of SATB1′s expression in 93 paired samples of rectal cancer and distant normal rectal tissue showed that SATB1 was significantly overexpressed on both the protein and mRNA levels in the cancer samples [94]. Additionally, SATB1′s expression correlated with TNM stage and the tumours’ invasion depth [94]. Moreover, in a panel of 5 human colon cancer cell lines, SATB1′s level was observed to be associated with the metastatic potential of the cells [94]. These results were next confirmed by Nodin et al., who assessed SATB1′s expression in CRC tumours from 529 patients and in 20 adjacent normal colon mucosa specimens [42]. SATB1 was found to be expressed in 42% of CRC cases, but not in non-malignant mucosa [42]. Furthermore, SATB1′s expression was found to be associated with microsatellite stable tumours and correlated with beta-catenin’s and SATB2′s levels [42]. SATB1′s overexpression in CRC samples as compared to adjacent non-malignant mucosa was further observed in numerous studies, including these by Jie Zhang et al. [50], Yi Zhang et al. [95] and many others [47,96,97,98,99,100]. To date, only one group has received different results [101]. Al-Sohaily with co-workers observed that SATB1′s level was significantly lower in the CRC samples of 352 patients than in paired non-malignant colon mucosa [101]. However, these different results could have been caused by the antibody cross-reactivity with the SATB2 protein, which is abundantly expressed in the normal colon mucosa.

In the majority of the abovementioned studies, SATB1 has been demonstrated to be a driver of malignant phenotype in CRCs. Its expression has been shown to be positively associated with invasion depth [94,95,96], poor degree of differentiation [47,50,95] and advanced TNM stage of the tumour [50,94,95,96]. An elevated SATB1 level was also connected to the presence of lymph node [95,96,98] and distant metastasis [95]. Moreover, SATB1′s expression was demonstrated to correlate with the atypical, cytoplasmic/nuclear expression patterns instead of membranous, β-catenin ones [47]. β-catenin is a key player in the Wnt/β-catenin pathway, which is thought to initiate EMT in CRCs [47]. In these tumours, β-catenin translocation from the cell membrane to the cytoplasm or nucleus may promote the expression of EMT-related proteins and pre-invasive factors [47]. It has been shown that SATB1 could play a dual role in CRCs, both as one of the Wnt/β-catenin pathway targets and as a regulator of β-catenin expression [102]. Furthermore, SATB1′s expression was demonstrated to be positively correlated with the expression of Vimentin, and negatively with the expression of E-cadherin and CK20 [47]. These findings indicate that SATB1 may be an important factor influencing EMT and metastasis in CRC tumours.

SATB1′s expression was also extensively studied in various CRC cell lines. Most of the CRC cell lines analysed were SATB1-positive [37,50,94,95,99,103]. Moreover, some studies additionally revealed an association between SATB1′s level and an aggressive, metastatic phenotype of the cells [50,94,95]. SiRNA-mediated SATB1′s knockdown in the highly metastatic LoVo cell line, which initially presented a high SATB1 expression level, caused decreased cell proliferation, lower ability to anchorage-independent growth, reduced invasion capability and a higher rate of apoptosis compared with the control [50]. Similar results from silencing SATB1 were also later observed in the RKO, LS147T, HT29 and HCT116 colorectal cancer cell lines [37,95,99]. In addition, SATB1′s depletion was shown to affect the expression of various metastasis-related proteins, including E-cadherin, N-cadherin, Slug, Twist1 and MMP7 (*Matrix Metalloproteinase-7*), which, at least partially, confirmed the impact of SATB1 on EMT and extracellular matrix degradation [37]; Table 1. In contrast, ectopic SATB1 overexpression in moderately differentiated SW480 cells resulted in a proliferation, colony formation rate, migration and invasion capability increment, as well as a decrease in apoptosis [95].

The above results were further confirmed in vivo in animal models. The injection of SATB1-depleted LS174T cells into mice dorsal flanks resulted in a significant reduction of the growth rate or even a total inhibition of the tumour growth, depending on the shRNA used [37]. On the other hand, unmodified LS174T cells grew rapidly, giving well-defined tumours [37]. Similarly, in an experiment using the gain-of-function model, ectopic SATB1′s overexpression in SW480 cells injected into mice resulted in a faster growth rate and an increase in the weight of the tumours compared to the control [95]. Additionally, SATB1′s expression in these cells promoted liver and lung metastasis [95].

Efforts to define the prognostic significance of SATB1′s expression in CRC tumours produced unclear results. On the one hand, there are numerous studies reporting SATB1′s expression in CRC as an independent factor of poor prognosis [95,96,98,100]. On the other hand, in some studies SATB1′s expression lacked a prognostic value [97] or was a prognostic factor only in SATB2-negative tumours [40]. Furthermore, it was even suggested that a high SATB1 level could be associated with a better OS [101]. However, a recent meta-analysis comprising data on SATB1′s expression in a cohort of 2083 CRC patients revealed that patients with a high SATB1 expression tended to have a shorter OS, and confirmed that a high SATB1 level was associated with a poor degree of tumour differentiation and the presence of distant metastasis [104]. Surprisingly, the analysis also showed that SATB1′s expression had no association with the tumour TNM stage [104].

To sum up, in numerous studies regarding CRCs, the SATB1 protein was demonstrated to be a factor promoting a malignant phenotype of the tumours, clearly associated with EMT, the metastasis process and a poor patient’s prognosis.

### 2.4. Prostate Cancer

Although there are only seven studies concerning SATB1′s expression in prostate cancer, they consistently show its role as a metastasis-promoting factor in this type of tumour. In 2013, Shukla et al. observed that SATB1 was significantly overexpressed in prostate tumours when compared to control tissues, and that its expression levels were associated with the grade of the tumour [43]. A couple of months later, similar results were reported by Mao et al., who showed that SATB1 was expressed in prostate cancer samples but not in the benign prostate hyperplasia [38]. Additionally, SATB1′s expression levels correlated with the Gleason score of the tumours, the presence of bone metastasis and the expression of the EMT markers [38]. A higher SATB1 level in prostate cancer specimens compared to benign samples was also observed by Qi et al. [46].

The cell culture studies revealed an elevated SATB1 expression in prostate cancer cell lines compared to normal prostate epithelial cells [43,46]. SATB1′s level was also shown to be positively associated with the aggressiveness of the cells [43] and their migration ability [38]. Additional studies on the loss-of-function models revealed that SATB1′s expression was required to maintain the invasive phenotype of prostate cancer cells. SATB1′s knockdown in prostate cancer cell lines (DU-145, PC-3M and LNCaP) significantly inhibited cell growth, proliferation and invasion rates [38,43,46,105]. Moreover, SATB1′s silencing in PC-3M cells was shown to increase E-cadherin’s expression, as well as to restore anchorage-dependent growth of the cells and their polarized morphology [43]. An augmented E-cadherin expression due to SATB1′s knockdown was also later observed in the DU-145 cell line [46]. In contrast, ectopic SATB1 overexpression in PZ-HPV-7 cells significantly decreased E-cadherin’s level and stimulated MMP-9′s (*Matrix Metalloproteinase-9*) expression [43]. Similarly, transfection with SATB1 expression plasmids was also shown to increase proliferation, migration, and the invasion capabilities of different prostate cancer cell lines [38,43,46].

These results were further validated in animal xenograft models. In 2013, Shukla and co-workers injected PC-3M-KO cells with stable SATB1 knockdown into dorsal flanks of nude athymic mice [43]. SATB1′s depletion was shown to significantly reduce both the weight and the size of the xenograft tumours [43]. Additionally, PC-3M-KO xenografts displayed a less aggressive phenotype and an increased E-cadherin expression compared to the control ones [43]. In two other studies, SATB1-depleted DU-145 cells were utilized as a loss-of-function model [68,106]. SATB1′s knockdown not only reduced the growth of the tumours, but also caused pyknosis of the cell nuclei and increased apoptosis [68,106]. In the gain-of-function model, SATB1′s overexpression in LNCaP cells was shown to stimulate the growth of the xenograft tumours and trigger EMT-promoting protein expression patterns [68]; Table 1.

The results of these studies strongly suggested an important role of SATB1′s expression in the progression of prostate cancer. Although its prognostic significance still needs to be evaluated, SATB1 seems to support the aggressive tumour phenotype and play a role in the stimulation of the EMT process and metastasis.

### 2.5. Gastric Cancer

The first attempts to define the clinicopathological and prognostic significance of SATB1′s expression in gastric cancer were made in 2010 by Lu et al. [107]. The authors observed a significant SATB1 overexpression in gastric tumours on both the protein and mRNA levels as compared to non-malignant tissue samples [107]. Furthermore, SATB1′s expression positively correlated with the depth of invasion, the TNM stage of the tumour and the presence of lymph nodes and distant metastasis [107]. Similar correlations between SATB1′s expression and patients’ clinicopathological data were also shown by Cheng et al. [108]. They observed a relationship between the level of SATB1 and the expression of Heparanase, an enzyme which is involved in basal membrane and extracellular matrix degradation. Hence, Heparanase could have a promoting role in gastric cancer spread, and therefore SATB1 could be associated with gastric cancer dissemination [108]. An elevated SATB1 expression in gastric cancer specimens, as well as its association with an advanced TNM stage and the presence of distant metastasis, was also observed by Yuan and colleagues [109]. Additionally, they revealed that SATB1′s expression was positively correlated with the HER2 level in gastric tumours [109].

All these studies clearly showed the prognostic value of SATB1′s expression in gastric tumours. The results were consistent, and SATB1′s overexpression in gastric tumours was associated with a significantly worse survival of the patients [107,108,109]. Further multivariable analyses identified SATB1′s expression as an independent negative prognostic factor [107,108]. However, all the studies only took into account the Chinese population, in which gastric cancer incidence and mortality are especially high, what resulted in numerous papers [110]. Some of them involved SATB1 as a potential therapeutic target. In 2014, Peng and co-workers used a novel thermosensitive magnetic system based on liposomes to co-deliver doxorubicin and SATB1 shRNA into the gastric cancer cells [111]. They observed a significant inhibition of the growth of the MKN-28 gastric cancer cell line in vitro [111]. These results were also confirmed in vivo in the mouse xenograft model [111]. Four years later another successful approach to anti-SATB1 targeted therapy was reported by Yang et al., who developed specific immunoliposomes targeting gastric cancer-initiating cells (CICs) to use them for SATB1-siRNA delivery [112]. Gastric CICs are highly aggressive “seeds” of gastric tumours considered to be responsible for cancer recurrence and metastasis. Therefore, their elimination could contribute greatly to gastric cancer treatment. So far, CICs-targeted SATB1 silencing has been tested in vitro and has generated promising results—SATB1′s knockdown reduced CICs proliferation by approximately 80% and decreased their population by approximately 60% [112]. Undoubtedly, more studies are needed to validate these results in vivo in the animal models as well. 

### 2.6. Other Neoplasms

The impact of SATB1′s expression on cancer progression and metastasis seems to be especially significant in the case of gastrointestinal tumours. Besides the earlier described colorectal and gastric cancers, SATB1 was also found to be associated with oesophageal cancer progression. It was demonstrated to promote oesophageal cancer cells growth and survival, and to be an independent negative prognostic factor in these tumours [113,114]. So far, SATB1 has been revealed to be a negative prognostic factor in all gastrointestinal cancers [115,116].

SATB1′s overexpression was also observed in liver [39,117] and pancreatic cancers. In hepatocellular carcinoma clinical samples, SATB1 level correlated with tumour size, a poor degree of differentiation and the presence of lymph node metastasis [39]. A similar relationship between SATB1′s expression and the presence of lymph nodes and distant metastasis was also seen in intrahepatic cholangiocarcinoma [117]. A high SATB1 level in in vitro studies was related to an aggressive phenotype of the cells [39,118]. Lastly, it was shown that in liver cancer SATB1 influenced the expression of more than one hundred genes related to tumour progression and metastasis, including genes coding for EMT-associated proteins such as Snail, Slug, Twist1, Vimentin and E-cadherin [39]; Table 1. In pancreatic cancer, SATB1 was found to be overexpressed and to promote cancer cell proliferation and invasion [119,120]. Moreover, its expression was strongly correlated with the tumour invasion depth and staging [119]. Finally, it was demonstrated that in pancreatic cancer an elevated SATB1 expression was associated with a shorter survival time of the patients [120,121].

The overexpression of SATB1 was noticed also in urinary bladder cancer cell lines and clinical samples [44,69,122]. Likewise, in this type of cancer, SATB1 was shown to induce EMT through the downregulation of E-cadherin and the upregulation of Snail, Slug and Vimentin [69]; Table 1. Not surprisingly, its expression was shown to correlate with the grade and stage of the tumours, as well as with the presence of lymph nodes and distant metastasis [44,69]. Additionally, an analysis of the loss-of-function and gain-of-function models revealed the influence of SATB1′s expression on bladder cancer cell proliferation, migration, apoptosis and sensitivity to cisplatin-based chemotherapy [44,69,122]. The fact that SATB1′s overexpression was shown to be associated with the shorter survival of bladder cancer patients [69] was not a surprise.

SATB1 was also demonstrated to play an important role in the progression of various gynaecological cancers. A significant SATB1 overexpression was observed in epithelial ovarian cancer cases as compared to normal ovarian tissue [40,48]. It was shown that the level of SATB1 was related to the FIGO stage of the tumour and to the presence of lymph node metastasis [48]. However, further research did not confirm these associations [40]. Despite that, in epithelial ovarian cancer SATB1′s expression was revealed to be a marker of poor prognosis [40,48]. SATB1 was shown to play a role in the development of endometrial carcinoma [28,123,124]. A significant overexpression was observed in endometrial cancer specimens in comparison to normal endometrial tissue [123,124]. Additionally, SATB1 expression level correlated with the grade of the tumour, its invasion depth, the TNM stage and the presence of lymph node metastasis [123,124]. Moreover, SATB1′s expression was an independent negative prognostic factor for endometrial cancer patients [124]. Similarly, an increased SATB1 expression and its correlation with the disease stage, the tumour grade and the presence of lymph node metastasis was also observed in cervical carcinoma [125]. In these tumours, a high level of SATB1 was observed to negatively affect both the overall and the disease-free survival of the patients [125]. 

## 3. Conclusions

Over the past 10 years the role of SATB1 in cancer progression has been extensively studied in the most common human neoplasms, i.e., breast, colon, lung, prostate and stomach cancers, and it has been described in more than 150 papers. There are also single reports available regarding less frequent neoplasms like choriocarcinoma or clear cell renal cell carcinoma. In the majority of the cancers studied, SATB1 was revealed to be a powerful factor influencing tumour invasion and metastasis. Its expression was associated with a poorly differentiated, aggressive phenotype of the tumours and a shortened patients’ survival. It was found to be a negative prognostic factor in numerous neoplasms, including breast [57], colorectal [104], gastric [107,108,109], pancreatic [120,121], ovarian [40,48], endometrial [124] and cervical cancers [125]. Many studies analysing the impact of SATB1 on EMT-related proteins’ expression demonstrated its role as an inductor of the mesenchymal phenotype of cancer cells [17,37,39,43,47]. In breast and colorectal cancers, SATB1 was shown to interfere with the Wnt/β-catenin pathway, which is thought to be crucial for metastasis [60,102,126]. Finally, silencing the protein impaired the growth of many cancer cells. In numerous loss-of-function models the expression of SATB1 was shown to be essential to maintain the invasive phenotype of cancer cells and their high proliferation rate [17,38,49,50].

However, in some subtypes of lung cancer an elevated SATB1 level was revealed to be a positive prognostic factor [91,92]. A loss of SATB1′s expression in these tumours was associated with a shorter overall survival of the patients [91,92]. A similar impact of SATB1′s expression on patients’ survival was also observed by Kowalczyk et al. in clear cell renal cell carcinoma samples. In this case, the positive impact of an elevated SATB1′s expression on the patients’ OS was shown to be an effect of miR-21-5p miRNA regulation [30]. Although some authors suggested a miRNA-mediated SATB1 regulation in lung cancer cells, additional studies are still needed in order to examine this more thoroughly [91]. Moreover, SATB1′s function and prognostic significance may vary in different lung cancer subtypes due to its heterogeneity.

Silencing SATB1′s expression with siRNA or shRNA was demonstrated to effectively supress cancer cell proliferation and invasion in vitro as well as in vivo in mice xenograft models [17,37,43,68,106]. These results may suggest that it could be a promising target for novel anticancer drugs. Baicalein and hydrophobic statins were shown to successfully down-regulate the level of SATB1 in breast and colorectal cancer cells respectively, causing a significant decrease in cells’ proliferation and invasion abilities [60,61,127]. The co-delivery of SATB1 shRNA and doxorubicin by using immunoliposomes was shown to have an antitumor effect on gastric cancer cells [111]. Similarly, therapy utilizing SATB1 shRNA to eliminate gastric cancer stem cells is also being intensively developed [112]. Finally, SATB1 was identified as a potential immune target for anti-cancer vaccines [128].

In conclusion, SATB1 is likely to become a target for many new experimental therapies in the nearest future. Due to its high prognostic significance in different tumours, it may also be considered as a molecular marker for novel diagnostic tests.

## Figures and Tables

**Table 1:** The impact of SATB1 on the expression of genes associated with EMT, metastasis, and cancer progression.

Category	Gene Symbol	Protein Name	Function	Effect of Modulation by SATB1	Malignancy
**Epithelial Markers**	*CDH1*	E-cadherin	Cell adhesion molecule, responsible for maintaining epithelial integrity [66]. Downregulation of E-cadherin, resulting in destabilization of adherens junctions, is one of the main hallmarks of EMT [67].	↓ Downregulation	Breast cancer [17] Colorectal cancer [37] Prostate cancer [43,46,68] Liver cancer [39] Bladder cancer [69]
	*CLDN1*	Claudin 1	Tight junctions protein that regulates the permeability of epithelia. During EMT, its expression is downregulated as a result of SNAIL, SLUG and Twist1 activity [67].	↓ Downregulation	Breast cancer [17]
**Mesenchymal Markers**	*CDH2*	N-cadherin	Cell adhesion molecule, taking part in various cellular processes, including proliferation, migration and apoptosis [70]. The gain of N-cadherin expression is thought to be a critical step in epithelial cancer metastasis [71].	↑ Upregulation	Breast cancer [17] Colorectal cancer [37]
	*VIM*	Vimentin	Intermediate filaments protein, expressed in mesenchymal cells. It maintains cell integrity and flexibility [72].	↑ Upregulation	Breast cancer [17] Prostate cancer [46,68] Liver cancer [39] Bladder cancer [69]
	*MMP-2, MMP-7, MMP-9*	Matrix metalloproteinases (MMPs)	A family of proteases that digest components of the extracellular matrix. MMPs not only allow cell migration, but can also contribute to EMT by activating TGFβ [73].	↑ Upregulation	Breast cancer [17] Colorectal cancer [37] Prostate cancer [43,68]
**EMT Inducers**	*TGFB*	Transforming Growth Factor β (TGF-β)	TGF-β is a multi-functional cytokine that regulates cell growth and differentiation, apoptosis, and cell motility [74]. TGF- β signalling has also been shown to play an important role in EMT induction [75].	↑ Upregulation	Breast cancer [17]
	*SNAI1*	SNAIL	Proteins belonging to the SNAIL superfamily of zinc-finger transcription factors. They repress E-cadherin expression and act as critical regulators of multiple pathways leading to EMT [76,77,78].	↑ Upregulation	Breast cancer [59] Liver cancer [39] Bladder cancer [69]
	*SNAI2*	SLUG		↑ Upregulation	Colorectal cancer [37] Liver cancer [39] Bladder cancer [69]
	*Twist1*	Twist1	TWIST1 is a basic helix-loop-helix (bHLH) transcription factor that plays a role of one of the most important EMT regulators [79,80]. It decreases E-cadherin, and increases N-cadherin expression. Furthermore, Twist1 can contribute to metastasis due to its pro-angiogenic and anti-apoptotic functions [80].	↑ Upregulation	Breast cancer [59] Colorectal cancer [37] Liver cancer [39]
**Other Factors**	*CCN2*	Connective Tissue Growth Factor (CTGF)	CTGF plays role in cells’ proliferation, adhesion, migration, and angiogenesis. It was also demonstrated to mediate tumorigenesis and increase metastatic potential of the cells [81,82].	↑ Upregulation	Breast cancer [17]
	*S100A4*	Metastasin	Ca-binding protein that regulates cell growth, survival and motility [83]. It was shown to increase cells’ migration and invasion capacities, and to be associated with tumour metastasis [84].	↑ Upregulation	Breast cancer [17]
	*VEGF-B*	VEGF B	A growth factor belonging to the vascular endothelial growth factor family. VEGF B is responsible for maintaining newly formed blood vessels, especially those developed under pathological conditions [85].	↑ Upregulation	Breast cancer [17]
